# Genome-Wide Identification of the Cation/Proton Antiporter (CPA) Gene Family and Functional Analysis of AtrNHX8 under Salt Stress

**DOI:** 10.3390/plants13121701

**Published:** 2024-06-19

**Authors:** Shengcai Liu, Zixian An, Yixuan Li, Rongzhi Yang, Zhongxiong Lai

**Affiliations:** 1 Institute of Horticultural Biotechnology, Fujian Agriculture and Forestry University, Fuzhou 350002, China; annzixian@163.com (Z.A.); 13231411695@163.com (Y.L.);; 2 Key Laboratory of Ministry of Education for Genetics, Breeding and Multiple Utilization of Crops, Fujian Agriculture and Forestry University, Fuzhou 350002, China

**Keywords:** *Amaranthus tricolor*, salt stress, *AtrCPA* gene family, *AtrNHX8*, functional analysis

## Abstract

*Amaranthus tricolor* is an important vegetable, and its quality is affected by salt stress. *Cation/proton antiporters* (*CPA*) contribute to plant development and tolerance to salt stress. In this study, 35 *CPA* genes were identified from a genome database for *A. tricolor*, including 9 *NHX*, 5 *KEA*, and 21 *CPA2* genes. Furthermore, in *A. tricolor*, the expression levels of most *AtrNHX* genes were higher at a low salinity level (50 or 100 mM NaCl) than in the control or 200 mM NaCl treatment. Levels of most *AtrNHX* genes were elevated in the stem. Moreover, *AtrNHX8* was homologous to *AtNHX4*, which is involved in the regulation of sodium homeostasis and salt stress response. After *AtrNHX8* overexpression in *Arabidopsis thaliana*, seed germination was better, and the flowering time was earlier than that of wild-type plants. Additionally, the overexpression of *AtrNHX8* in *A. thaliana* improved salt tolerance. These results reveal the roles of *AtrNHX* genes under salt stress and provide valuable information on this gene family in amaranth.

## 1. Introduction

*Amaranthus tricolor* L. is an important vegetable containing betalains, carotenoids, alkaloids, and chlorophylls. It exerts anti-oxidative, anti-cancer, anti-viral, anti-parasitic, and radical-scavenging effects and may be useful for treating certain oxidative stress-related disorders [1,2,3]. It can be cultivated in diverse environmental conditions because it is highly resistant to environmental stresses [4,5,6,7], such as salinity, temperature, and drought stress.

Salt-affected cultivated lands account for a large proportion of global farmland, with more than one-third of irrigated land exhibiting some degree of salinization [8]. Salt stress has become one of the most serious abiotic stresses because it can impair soil fertility and crop growth and development substantially [9], affecting crop yield and quality as well as agricultural sustainability [10,11]. When plants are exposed to salt stress, they must constantly adjust their cellular ion contents to maintain K^+^ and Na^+^ homeostasis [12]. The homeostasis of cations and pH by exchanging Na^+^, K^+^, or Li^+^ for H^+^ is regulated by monovalent cation–proton antiporters (CPAs) in the process of salt stress [13]. CPAs can be classified into two categories: The CPA1 family (including Na^+^/H^+^ NHX antiporters) and the CPA2 family (including Cation/H^+^ (CHX) and K^+^ efflux antiporters (KEA)). The NHX type is a particularly important CPA1 exchanger involved in Na^+^ transport and therefore salinity tolerance [9].

The *NHX* gene family includes eight members (AtNHX1–8) in *Arabidopsis* [14]. AtNHX1–4, AtNHX5–6, and AtNHX7–8 can be separated into the vacuole (Vac) subfamily, plasma membrane (PM) subfamily, and endosome nucleolus (Endo) subfamily, respectively, according to their subcellular localization [15,16,17]. *NHX* genes may play an important role in the salt stress response and salt tolerance [9]. Under salt stress, NHXs may prevent Na^+^ in the vacuoles of most tissues from entering the cytosol [18]. They regulate pH and Na^+^ homeostasis in the cell to reduce the toxic concentration of Na^+^ in the cytosol [19]. Studies have reported the responses of various *NHX* genes to salt stress, such as *CmoNHX1* [9] and *GbNHX2* [20]. *BvNHX5* may interact with CBL and CIPK to enhance salt tolerance in *Beta vulgaris* [21]. *PgNHXs* could play significant roles in the response to salt stress in *Punica granatum* [22]. The *LeNHX* gene shows increased expression in tomato under salt stress [23]. The overexpression of *AtNHX1* in *Arabidopsis* could confer salt tolerance under NaCl stress. The overexpression of *OsNHX1* in rice promotes root growth and water uptake under low salt stress [24]. Upon treatment with 300 mM NaCl, the transcript levels of *AtNHX4* increased initially and then decreased gradually [25].

In the study, we screened and identified members of the AtrCPA gene family from the *Amaranthus tricolor* genome database. *AtrCPA*s were comprehensively characterized. The NHX type is a particularly important CPA1 exchanger; accordingly, the expression levels of *AtrNHX* genes were evaluated under salt stress by qRT-PCR. Finally, the effects of the overexpression of *AtrNHX8* were evaluated in *Arabidopsis thaliana* under salt stress. The results reveal the roles of *AtrNHX* genes in the response to salt stress and provide a valuable reference to further explore the functions of *NHX* genes in amaranth.

## 2. Results

### 2.1. Identification of Cation–Proton Antiporter Gene Family Members and Analyses of Physicochemical Properties

In total, 35 CPA genes were screened from the Amaranthus genome database [26], including 9 *NHXs*, 5 *KEAs*, and 21 *CPA2s*. All of the genes were renamed according to the order of gene sequence IDs (Table 1). The molecular weight ranged from 25.08 kDa (AtrCPA2-7) to 135.36 kDa (AtrKEA3). The theoretical isoelectric point ranged from 4.58 (AtrCPA2-17) to 9.47 (AtrCPA2-20). All AtrNHX proteins were hydrophobic according to the grand average of hydropathy (GRAVY) value. Only AtrKEA3 and AtrKEA5 had a signal peptide. Thirty-four CPAs had typical transmembrane helix regions, other than AtrKEA2 (Supplementary Figure S1). The prediction of subcellular localization patterns showed that AtrNHX proteins may be localized in the plasma membrane or vacuole. The detailed physical and chemical properties are shown in Table 1.

### 2.2. Motif Composition, Conserved Domains, and Gene Structure

**Figure 1:**
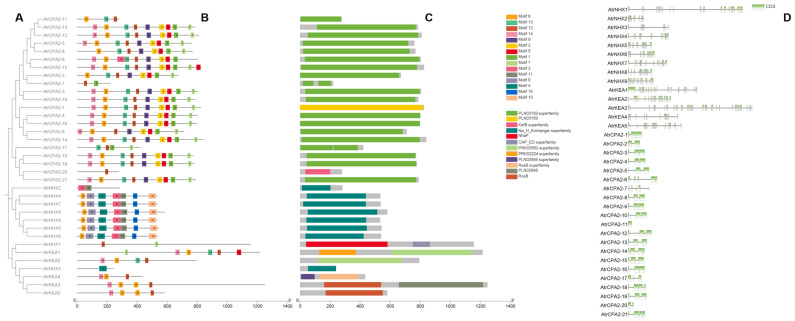
Conserved motifs and gene structure of *AtrCPAs*. (**A**) Phylogeny analysis of *AtrCPAs*; (**B**) conserved motif distribution in AtrCPA proteins; (**C**) conserved domains of AtrCPA proteins; (**D**) exon–intron structure of *AtrCPAs*. TBtools v. 2.086 was used to present the chart.

Thirty-five *AtrCPA* members were divided into three subfamilies, and most *AtrCPA* members in the same subfamily had similar motif compositions and domains, suggesting that these proteins have similar functions (Figure 1A–C). Motif 8 was found in all three subfamilies; its conservation suggests that it may be useful for the identification of CPA-type proteins in *A. tricolor*. Each subfamily had unique conserved moftis. Motifs 3, 4, 6, 10, 11, and 15 were conserved in NHX-type proteins. Motifs 8, 12, and14 were conserved in the *KEA* subfamily. Motifs 1, 2, 9, and 12 were conserved in CPA2-type proteins as well. The numbers and lengths of exons and introns differed among the three subfamilies of *AtrCPAs* (Figure 1D). The gene structures of *AtrNHXs* and *AtrKEA*s were relatively complex, while the structure was relatively simple in the *AtrCPA2* subfamily.

### 2.3. Promoter Element Analysis

**Figure 2:**
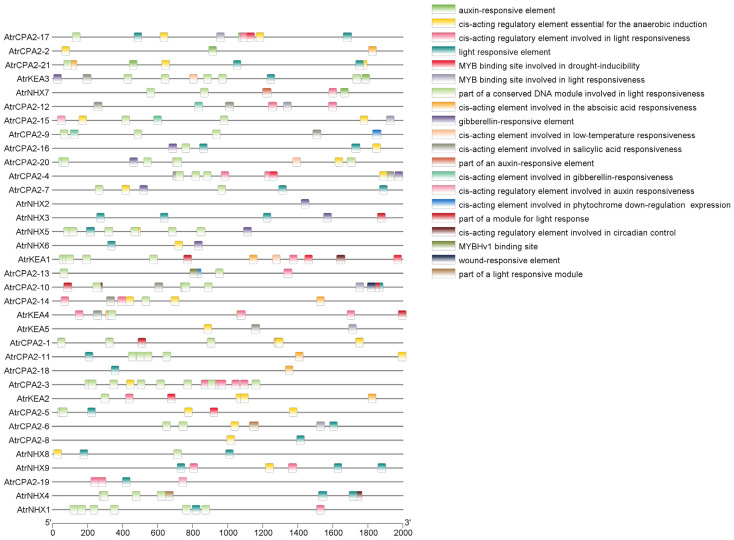
Distribution *cis*-acting regulatory elements in the promoter regions of *AtrCPAs*. TBtools v. 2.086 was used to present the chart.

Putative promoter sequences of *AtrCPAs* were searched against PlantCARE to identify potential *cis*-acting regulatory elements, with a focus on hormone- and stress-related *cis*-acting elements (Figure 2). A total of 41 kinds of *cis*-acting elements were identified, suggesting that the expression of *AtrCPAs* is regulated by various mechanisms. Additionally, 162 *cis*-acting elements were related to hormones, including abscisic acid (ABA), auxin (IAA), gibberellin (GA), salicylic acid (SA), and methyl jasmonate (MeJA), and 501 *cis*-acting elements and binding sites were related to stress, including low temperature, drought, wound, and anaerobic conditions. Among these elements, the contents of antioxidant- and defense-related elements were high.

### 2.4. Chromosomal Localization and Synteny Analysis of AtrCPAs

**Figure 3:**
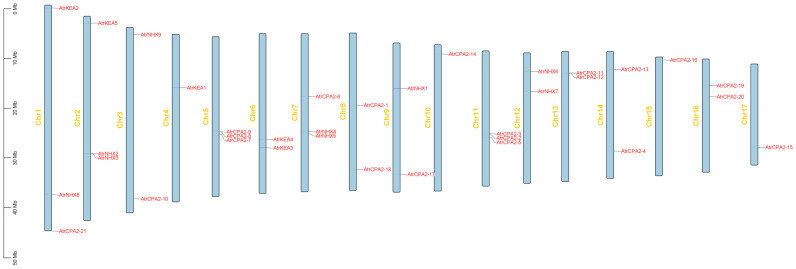
Chromosomal distribution of 35 *AtrCPA* genes.

Based on the location data from the *Amaranthus tricolor* genome database, 35 *AtrCPAs* were mapped to 17 chromosomes, with an uneven distribution (Figure 3). Chr1, Chr 2, Chr 5, Chr 7, and Chr 11 each contained three *AtrCPA* members.

**Figure 4:**
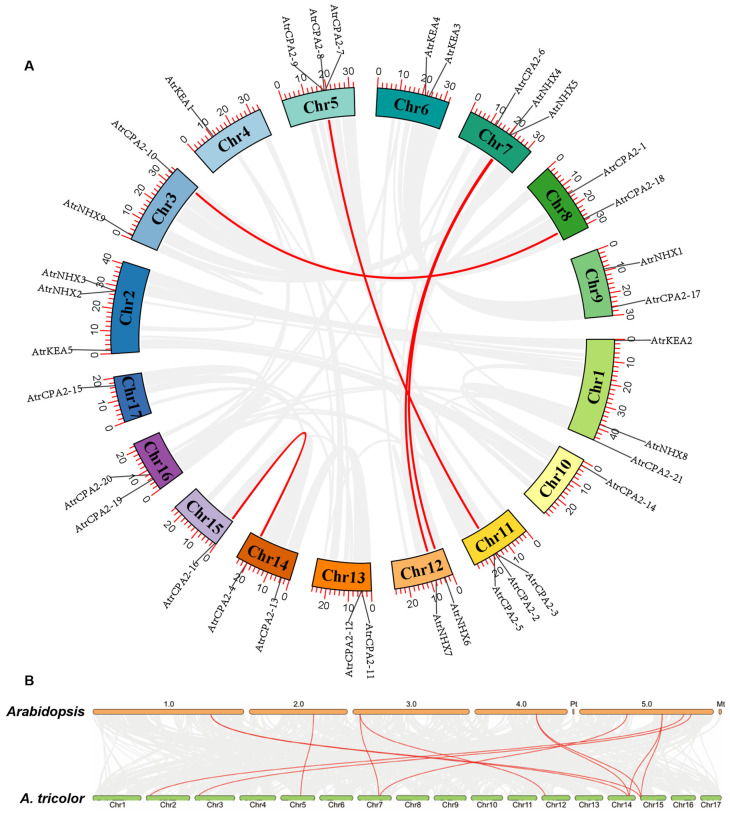
Syntenic analyses of *CPA* genes. (**A**) Segmental duplication events of *CPA* genes in *Amaranthus tricolor*. (**B**) Duplication events of *NHX* genes in *Amaranthus tricolor* and *Arabidopsis*. Red lines indicate *CPA* duplication events. Gray lines represent all synteny blocks. TBtools v. 2.086 was used to present the chart.

Nine segmental duplication events involving ten *AtrCPA* genes were filtered out. No tandem duplication event was found in the *AtrCPA* genes (Figure 4A). To further study the origin and divergence of *AtrCPA* genes, duplication events were evaluated by comparisons between *Amaranthus tricolor* and *Arabidopsis*. A total of 12 collinear *CPA* gene pairs between *Arabidopsis* and *Amaranthus tricolor* were identified (Figure 4B). Only *AtrNHX5*, *AtrNHX6*, *AtrNHX9*, *AtrKEA5*, *AtrCPA2-4*, *AtrCPA2-9*, and *AtrCPA2-16* had homologues in *Arabidopsis*, suggesting that other genes arose after the divergence of *Arabidopsis*.

### 2.5. Protein Interaction Analysis

**Figure 5:**
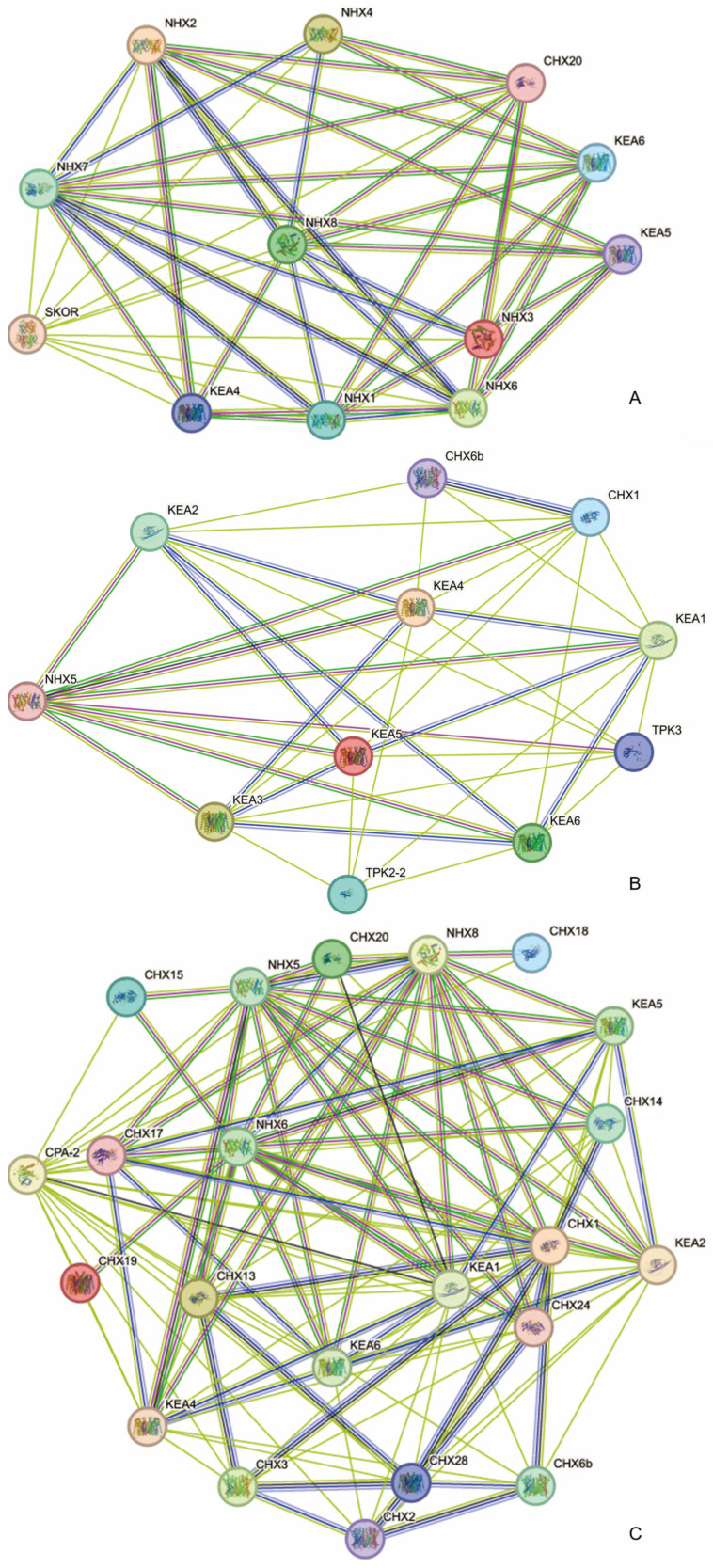
Protein–protein interaction networks involving CPAs in *Arabidopsis*. (**A**–**C**). Interaction networks of AtNHXs (**A**), AtKEAs (**B**), and AtCPA2 (**C**).

To predict which proteins interact with CPAs, we used the STRING database to construct interaction networks involving *Arabidopsis* CPA proteins (Figure 5A–C). In the *NHX* subfamily, the NHX7 and NHX8 proteins showed a strong interaction with other NHX proteins in *A. thaliana* (Figure 5A). In the *KEA* subfamily (Figure 5B), the KEA2 and KEA3 proteins were predicted to interact closely with other KEA proteins in *A. thaliana.* In the *CPA2* subfamily (Figure 5C), the CPA-2 and NHX8 proteins were predicted to interact closely with other KEA, CPA2, and NHXs in *A. thaliana*.

### 2.6. Phylogenetic Analysis of NHXs

**Figure 6:**
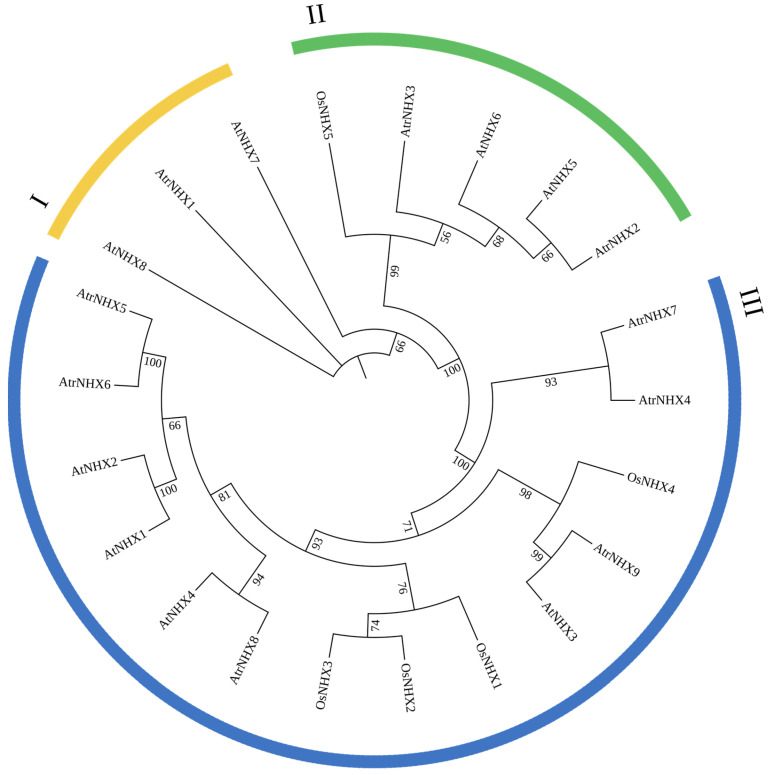
Neighbor-joining phylogenetic tree of NHX proteins from *Amaranth tricolor*, *Arabidopsis thaliana*, and *Oryza sativa.* NHXs were divided into three major groups. The phylogenetic tree was constructed using MEGA X. The numbers at the nodes indicate bootstrap 1000.

Because *NHX* genes may play an important role in the salt stress response and salt tolerance [9], we focused on this sub-family. NHX proteins with full-length sequences from *Arabidopsis thaliana* and *Oryza sativa* were used to construct a phylogenetic tree based on AtrNHX proteins (Figure 6). The NHX proteins were divided into three clades, clades I, II, and III, with strong support (Bootstrap = 100%). AtrNHX1 was closely related to AtNHX7 and AtNHX8 in *Arabidopsis*, assigned to clade I; AtrNHX2 and AtrNHX3 were closely related to AtNHX5 and AtNHX6 in *Arabidopsi*s, assigned to clade II; and other AtrNHXs were assigned to clade III, indicating that these genes share similar functions. In particular, *AtrNHX8* was closely related to *AtNHX4*, which is involved in the regulation of sodium homeostasis and salt stress response.

### 2.7. qRT-PCR Analysis of AtrNHXs under Salt Stress

**Figure 7:**
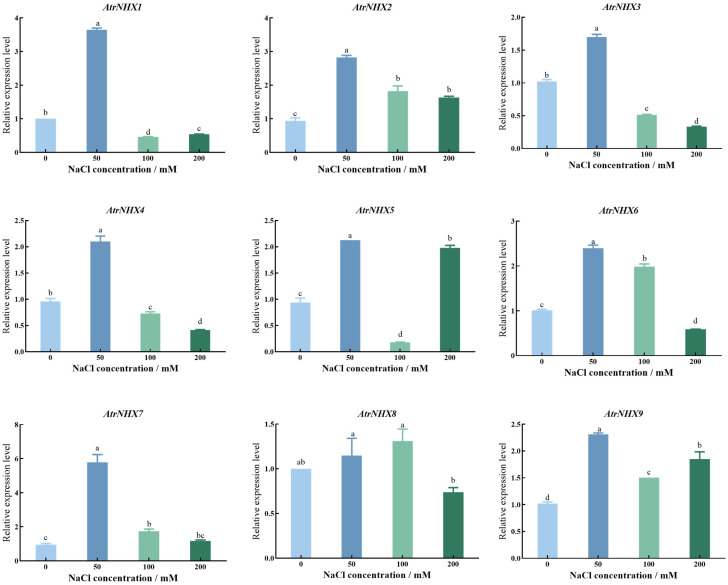
qRT-PCR analysis of *AtrNHX* genes under salt stress. The data are presented as mean ± standard error and were subjected to analysis of variance (ANOVA). The means were compared using the ad hoc Tukey test (*p* < 0.05%). Lowercase letters represent significant differences at the 0.05 level.

The relative expression patterns of *AtrNHX* genes were analyzed (Figure 7). At a low salinity level (50 mM NaCl), the expression levels of *AtrNHX* genes were higher than those in the control or 200 mM NaCl groups, other than *AtrNHX1* and *AtrNHX2*, suggesting that a low salinity level is favorable for amranth growth. The expression levels of *AtrNHX2*, *AtrNHX4*, *AtrNHX6*, and *AtrNHX8* were higher under 100 mM NaCl than in the control. These results indicated that the genes play a key role in tolerance to moderate salt stress in *A. tricolor*. 

### 2.8. Analysis of AtrNHX Gene Expression in Different Tissues of Amaranth

**Figure 8:**
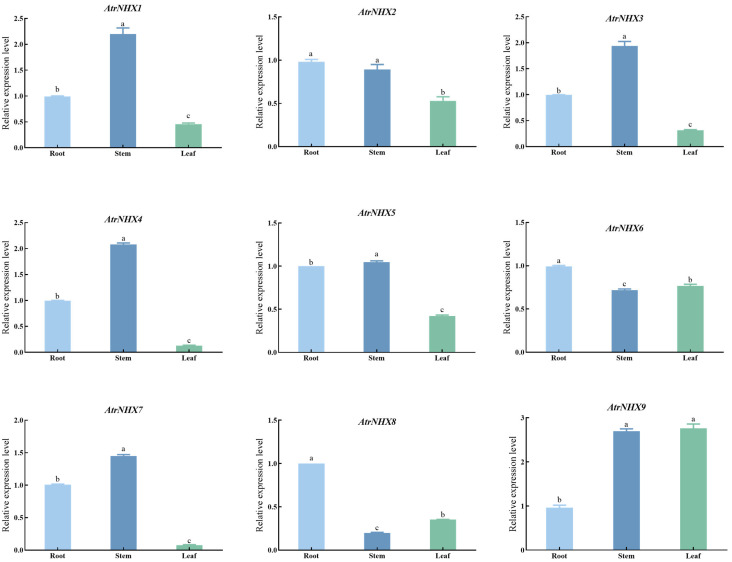
Expression trends of *AtrNHX* genes in different tissues. The data are presented as mean ± standard error and were subjected to analysis of variance (ANOVA). The means were compared using the ad hoc Tukey test (*p* < 0.05%). Lowercase letters represent significant differences at the 0.05 level.

A qRT-PCR analysis of *NHX* genes in different tissues of amaranth indicated that the relative expression levels of *AtrNHX9* in leaves were higher than those in the roots. *AtrNHX2*, *AtrNHX3*, *AtrNHX4*, *AtrNHX5*, *AtrNHX7*, and *AtrNHX9* levels in the stem were higher than those in the root. *AtrNHX6* and *AtrNHX8* showed high expression in the root (Figure 8). These results showed that *AtrNHXs* play different roles in different tissues of *A. tricolor*.

### 2.9. Overexpression of AtrNHX8 Enhances Salt Tolerance in Arabidopsis

**Figure 9:**
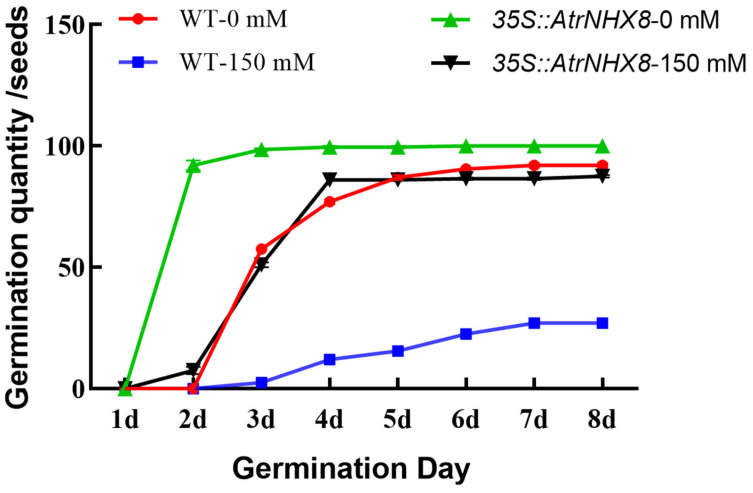
Seed germination in *Arabidopsis thaliana* under salt stress.

The seed germination rate of *35S::AtrNHX8* was significantly higher than that of the wild type (WT) under 150 mM NaCl treatment. Furthermore, seed germination in *35S::AtrNHX8* was significantly earlier compared with that of WT under NaCl treatment (Figure 9). Germination was nearly complete in *35S::AtrNHX8* and WT after 2 days and 6 days, respectively. 

**Figure 10:**
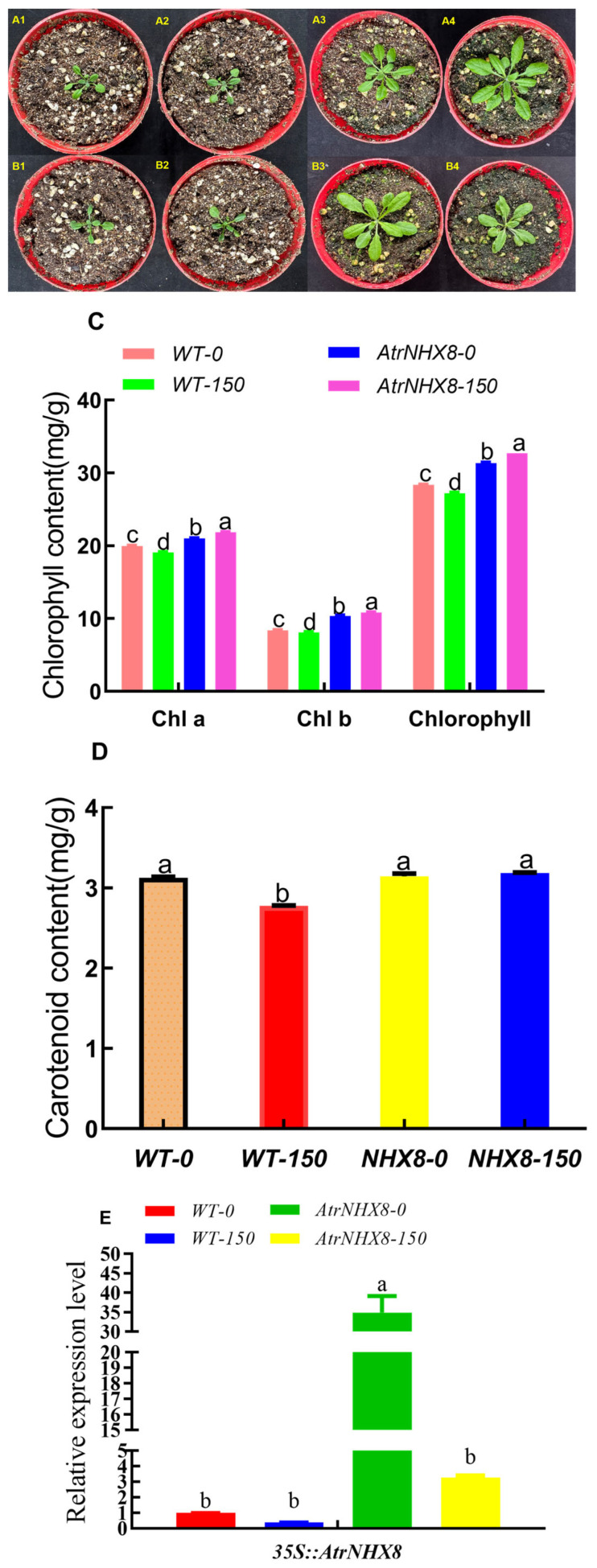
Relative expression of *GUS* in *Arabidopsis thaliana* leaves under salt stress. (**A1**,**A2**) represent wild-type *Arabidopsis* before salt treatment, (**A3**,**A4**) represents wild-type *Arabidopsis* with 0 mM and 150 mM NaCl treatment for 7 days, respectively. (**B1**,**B2**) represent *35S::AtrNHX8 Arabidopsis* before salt treatment, (**B3**,**B4**) represent *35S::AtrNHX8 Arabidopsis* with 0 mM and 150 mM NaCl treatment for 7 days, respectively. (**C**,**D**) show the chlorophyll and carotenoid contents, respectively. (**E**) represents the relative expression of *NHX8* in *Arabidopsis thaliana* leaves. The data are presented as mean ± standard error and were subjected to analysis of variance (ANOVA). The means were compared using the ad hoc Tukey test (*p* < 0.05%). Lowercase letters represent significant differences at the 0.05 level.

To analyze the salt response in adult plants, 15-day-old seedlings in pots were irrigated with 150 mM NaCl solutions or water as a control. The phenotypic effects of salt treatment for 7 days are shown in Figure 10. The plant height and leaf size under salt stress in WT and *35S::AtrNHX8* lines were shorter and smaller compared with those under non-salt stress. There were fewer leaves with salt stress than without salt (Figure 10A,B). The contents of chlorophyll a, chlorophyll b, and total chlorophyll in WT *Arabidopsis thaliana* under salt stress were significantly (*p* ≤ 0.05) lower than those without salt stress. However, the opposite results were obtained for *35S::AtrNHX8* plants. Furthermore, the chlorophyll content in *35S::AtrNHX8* plants was significantly (*p* ≤ 0.05) higher than that in the WT (Figure 10C). The carotenoid content in *35S::AtrNHX8* plants was significantly (*p* ≤ 0.05) higher than that in the WT under salt stress (Figure 10D).

*AtrNHX8* gene expression levels in *Arabidopsis thaliana* leaves were analyzed. The expression of the *AtrNHX8* gene was significantly higher in WT *Arabidopsis* and *35S::AtrNHX8* transformed plants under salt stress than under non-salt stress (Figure 10E).

### 2.10. AtrNHX8 Could Promote Flowering in Arabidopsis thaliana

**Figure 11:**
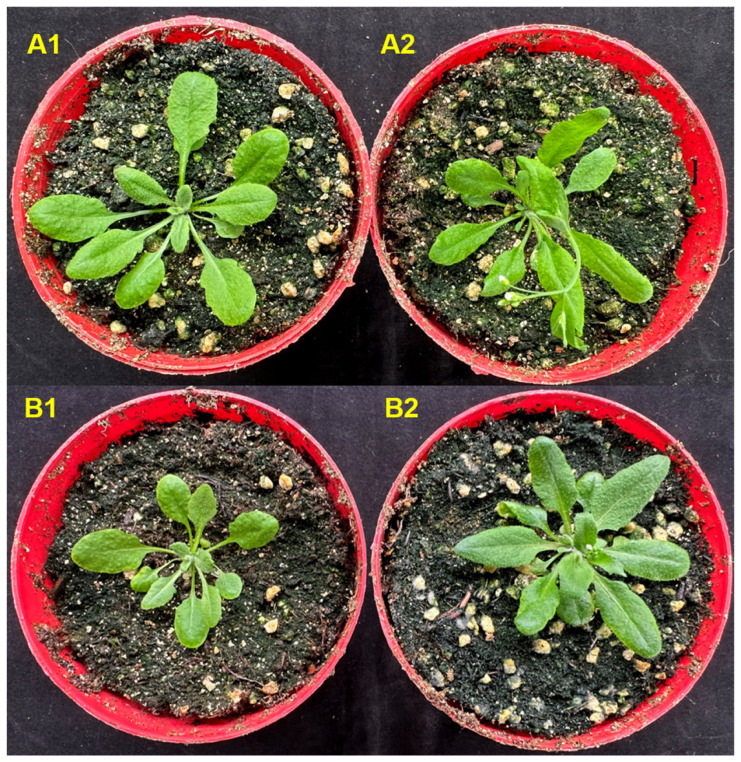
Flowering diagram of wild-type and *AtrNHX8* transgenic plants. (**A1**,**A2**) represent wild-type *Arabidopsis thaliana* and *35S::AtrNHX8* lines without salt treatment; (**B1**,**B2**) represent wild-type and *35S::AtrNHX8* lines with 150 mM NaCl. *AtrNHX8* promoted flowering in *Arabidopsis thaliana*.

Under salt stress, *35S::AtrNHX8* lines at the 10-leaf period began to flower after 9 days. However, WT plants at the 21-leaf period began to flower after 18 days. Without salt stress, *35S::AtrNHX8* lines at the 10-leaf period began to flower after 9 days of treatment. WT plants at the 16-leaf period began to flower after 13 days of treatment (Figure 11). The results indicated that *AtrNHX8* could promote flowering in *Arabidopsis thaliana*.

## 3. Discussion

CPAs can be divided into two categories: The CPA1 family, which includes Na^+^/H^+^ NHX antiporters, and the CPA2 family, which includes CHXs and KEAs. They play key roles in abiotic stress tolerance, especially in responses to salt stress [27]. The CPA family has been characterized in several plant species, such as *Arabidopsis thaliana* [28], *Oryza sativa* [29], *Brassica napus* [30], and *Raphanus sativus* [31]. In the present study, 35 CPA genes were identified in the genome of *A. tricolor*, including 9 NHX genes, 5 KEA genes, and 21 CPA2 genes. These CPA gene counts differed from those in other species, indicating that the CPA gene family underwent duplication events and expansions. A subcellular localization analysis of AtrCPA implied they play multiple functions in plants. 

Gene functions can be predicted based on comparisons with previously characterized orthologues [32,33]. Gene evolution and potential functional differences were involved in the differences in conserved motifs. Additionally, a phylogenetic analysis showed that the nine members of the NHX family in *A. tricolor* could be divided into three subfamilies. *AtrNHX9* was paired with *AtNHX3* in subclade B1. Previous studies have shown that *AtNHX3* plays a role in maintaining potassium ion homeostasis during germination and seedling growth in *Arabidopsis* [34]. *AtrNHX8* was closely related to *AtNHX4*, which is involved in the regulation of sodium homeostasis and the salt stress response. These results verified that orthologous proteins have similar functions and that the *NHX* family genes are relatively conserved. 

qRT-PCR analyses of *AtrNHX* gene family members under salt solution treatment revealed different gene expression trends. As the salt concentration increased, gene expression levels increased and then decreased, consistent with the results of previous studies [35]. Upon treatment with 300 mM NaCl, the transcript levels of *AtNHX4* increased and then gradually decreased; quantitative real-time PCR analyses showed similar expression patterns [25]. However, the knock-out of *AtNHX4* could enhance tolerance to salt stress, and Na^+^ contents under high NaCl stress were lower than those in WT plants [25]. *AtrNHX2* and *AtrNHX8* had the highest expression under 100 mM NaCl, and the remaining members were all expressed at 50 mM NaCl. There were differences in the responses of the *AtrNHX* genes under salt stress. A qRT-PCR analysis revealed that the family members were differentially expressed in various parts, with most family members expressed in the roots and stems, consistent with a previous study showing that *NHX* genes are highly expressed in the roots under salt stress, thereby enhancing salt tolerance [36]. The results of this study provide a basis for further exploration of the *NHX* gene family and provide a reference for analyses of the mechanism by which *NHX* genes contribute to the growth of amaranth, responses to abiotic stress, and development in amaranth.

In this study, salt stress inhibited seed germination in amaranth, while *35S::AtrNHX8 Arabidopsis* plants showed an increased seed germination rate, earlier germination, and improved salt tolerance. A previous study has shown that salt stress delayed seed germination and reduced the germination rate in *Medicago sativa,* and the germination rate and germination potential of transformed plants were significantly higher than those of WT plants under salt stress conditions [11]. 

Both WT *Arabidopsis* and *35S::AtrNHX8* transformed plants were smaller and showed a lower leaf area and leaf number under the salt stress than in the non-salt stress treatment. Furthermore, the flowering time of the transformed plants was earlier than that of WT *Arabidopsis*. *35S::AtrNHX8*-transformed plants showed higher chlorophyll contents under salt stress treatment than under non-salt stress. However, WT plants showed lower chlorophyll contents under salt stress conditions than under non-salt stress. Salt stress resulted in a decrease in the chlorophyll content in *Typha domingensis* Pers [37], consistent with our results.

*NHX* genes in the CPA (monovalent cationic reversal protein) family are widely distributed in bacteria, fungi, and higher plants and animals. The family is involved in the regulation of the cell cycle and proliferation, salt tolerance, vesicle trafficking, and growth [38]. Furthermore, *NHX* genes regulate flower coloration [39,40,41] and flower development [42]. The results of this study indicate that *AtrNHX8* could promote flowering in *Arabidopsis thaliana*. Further studies are needed to determine the mechanism underlying the effects of *AtrNHX8*. 

## 4. Materials and Methods

### 4.1. Identification of and Characterization NHX Gene Family Members in Amaranthus tricolor

Genome data for amaranth were downloaded from http://ftp.agis.org.cn:8888/~fanwei/Amaranthus_tricolor/ [26]. Gene screening was performed using a hidden Markov model. The NHX domain was downloaded from Pfam 36.0 (PF00999) (http://pfam.xfam.org/; accessed on 19 October 2021), and HMMER was used to identify all possible *NHX* genes in amaranth [43] with an e-value ≤ 5. Each candidate NHX gene was further confirmed using the Conserved Domain Database (CDD v3.21, http://www.ncbi.nlm.nih.gov/Structure/cdd/wrpsb.cgi). Physical parameters of the deduced NHX proteins were investigated using ExPASy (http://web.expasy.org/protparam/). 

Euk-mPLoc 2.0 (http://www.csbio.sjtu.edu.cn/bioinf/euk-multi-2/) was used to evaluate the subcellular localization of *AtrCPAs*. Trans-membrane domains were identified using the TMHMM Server v. 0.2.0 (http://www.cbs.dtu.dk/services/TMHMM/).

### 4.2. Gene Structure, Motif Composition, and Promoter Analyses 

TBtools (v. 2.086, Guangzhou, China) [44] was used to analyze the exon–intron organization of AtrCPAs. Based on the amaranth genome, 2000 bp sequences upstream of the start codon (ATG) of *AtrCPA* genes were acquired. PlantCARE (http://bioinformatics.psb.ugent.be/webtools/plantcare/html/) [45] was used to predict *cis*-acting regulatory elements with default parameters. Conserved motifs of AtrNHX proteins were identified using the online MEME (Multiple Expectation Maximization for Motif Elicitation) (http://meme-suite.org/tools/meme (accessed on 29 October 2022). Parameter settings were set as follows: maximum number of motifs, 15; optimum motif width, ≥6 and ≤50.

To retrieve and display the repeatedly detected association networks, protein sequences were submitted to STRING (Search Tool for Recurring Instances of Neighboring Genes) v. 11.0 (https://cn.string-db.org/).

### 4.3. Chromosomal Localization and Synteny Analyses

The GFF3 file that contained location data for amaranth *CPA* genes was downloaded from the *Amaranthus tricolor* Genome Database [26]. The chromosomal location and results of the synteny analysis for *NHX* family genes were visualized using TBtools software (v. 2.086). The *Arabidopsis thaliana* genome was downloaded from TAIR for a synteny analysis with the amaranth genome. 

### 4.4. Sequence Alignment and Phylogenetic Analysis of NHXs

A multiple sequence alignment of the full-length amino acid sequences of NHXs from *Amaranthsus tricolor*, *Arabidopsis thaliana*, and *Oryza sativa* was generated using MUSCLE (Multiple Protein Sequence Alignment) within MEGA (Molecular Evolutionary Genetics Analysis) v. 11 (https://www.megasoftware.net/). Subsequently, a phylogenetic tree was constructed using the neighbor-joining method (NJ) using MEGA11 (Version 11.0.8) with 1000 bootstrap replicates, the Jones–Taylor–Thornton (JTT) model, and pairwise deletion. The NHX protein sequences in *Arabidopsis thaliana* and *Oryza sativa* were downloaded from the *Arabidopsis* Information Resource (TAIR) database (http://www.arabidopsis.org/) and https://ricedata.cn, respectively.

### 4.5. Plant Material, Treatment, and qRT-PCR Analysis

Amaranth seeds of “Suxian No. 1” (supplied by the Suzhou Academy of Agricultural Sciences) were sown in culture pots in a chamber at 25 °C for 16/8 h (day/night). At the 3-leaf seedling stage, they were treated with different concentrations of NaCl (0, 50, 100, and 200 mM) in three biological replicates. The seedlings were grown in the same chamber at 25 °C and 16/8 h (D/N). The amaranth leaves were collected after 7 days of salt solution treatment. All samples were immediately frozen in liquid nitrogen and stored at −80 °C.

Total RNA was isolated from the collected samples using a kit (Yeasen, Shanghai, China) according to the manufacturer’s instructions. First-strand cDNA was synthesized from 1 μg of total RNA using Recombinant M-MLV Reverse Transcriptase (TransGen Biotech, Beijing, China). Quantitative real time-PCR (qRT-PCR) was performed in optical 96-well plates using the Roche LightCycler 480 instrument (Roche, Solna, Sweden). The reactions were carried out in a 20 μL volume containing 10 μL of SYBR Premix Ex Taq, 0.8 μL of specific primers, 2 μL of diluted cDNA, and 6.4 μL of ddH_2_O. The PCR conditions were as follows: 30 s at 95 °C, 45 cycles of 10 s at 95 °C, and 20 s at 59 °C, followed by 12 s at 72 °C. *AtrEF1a* [46] was used as the internal reference gene. The 2^−ΔΔCt^ quantification method was used, and variation in expression was estimated from the three biological replicates. The primer pairs used for the qRT-PCR analysis of *NHX* genes are listed in Table 2.

### 4.6. Functional Analysis of AtrNHX8

The *AtrNHX8* gene was amplified by PCR and ligated to the expression vector pCambia1301-35S-GUS. Then, the constructed recombinant vector pCambia1301-35S-*AtrNHX8*-GUS was transferred into *Agrobacterium tumefaciens* GV3101 by the freeze–thaw method [47]. In addition, transgenic *A. thaliana* plants were obtained by the floral dip method [48]. T2-generation transgenic plants were finally obtained through multi-generation selfing, GUS staining, and PCR. The primers used for vector construction and PCR detection are listed in Table 3.

To better understand the mechanism by which *AtrNHX8* responded to salt stress, WT and T2 generation transgenic seeds from *Arabidopsis* were placed in Petri dishes with 3-layer filter paper, and 15 mL of 150 mM NaCl was added to each dish. Water was used as the control. In all cases, experiments were carried out in (at least) triplicate. The germination potential and final germination rate were determined at 3 days and 7 days. 

The WT and T2 generation transgenic *Arabidopsis* seeds were sown in culture pots. Plants at the 6-leaf stage were treated with 150 mM salt solution. After 7 days of salt treatment, the leaves of WT and transgenic *Arabidopsis thaliana* were collected to extract RNA for qRT-PCR, following the previously described method. The primers for the *AtNHX* gene used for qRT-PCR are listed in Table 3. The chlorophyll and carotenoid contents were evaluated according to Liu [2]. 

### 4.7. Statistical Analyses

Data were analyzed using SPSS 17.0 software. Values of *p* < 0.05 were significant in comparisons between the treatments and controls. GraphPad Prism 6.01 was used to generate histograms. Figures were generated using TBtools.

## 5. Conclusions

Nine *NHX* genes were identified from a full-length transcriptome database for *A. tricolor*. *AtrNHX8* was homologous to *AtNHX4*, which is involved in the regulation of sodium homeostasis and the salt stress response. *AtrNHX8* overexpression in *Arabidopsis thaliana* resulted in better seed germination and earlier flowering times than those of the WT.

## Figures and Tables

**Table 1:** Identification and sequence analysis of the CPA gene family in amaranth.

Sequence ID	Gene Name	Number of Amino Acids	Molecular Weight/kDa	Theoretical pI	Instability Index	Aliphatic Index	Grand Average of Hydropathicity	Number of Predicted TMHs	Localization
g1127.t1	*AtrNHX1*	1158	128.23	6.96	36.53	98.32	0.051	12	Plasma Membrane
g3986.t1	*AtrNHX2*	283	31.85	5.57	54.17	97.49	0.195	5	Vacuole
g3987.t1	*AtrNHX3*	241	25.90	5.46	43.79	123.4	0.834	5	Vacuole
g6525.t1	*AtrNHX4*	536	59.05	6.08	37.48	114.94	0.515	11	Plasma Membrane
g6555.t1	*AtrNHX5*	544	60.34	7.73	30.13	107.5	0.55	11	Vacuole
g14299.t1	*AtrNHX6*	540	59.74	6.31	32.79	107.04	0.517	12	Vacuole
g14654.t1	*AtrNHX7*	538	59.16	6.56	33.61	113.98	0.538	11	Plasma Membrane
g21099.t1	*AtrNHX8*	535	59.64	7.03	42.28	109.1	0.534	11	Vacuole
g25914.t1	*AtrNHX9*	582	65.77	8	33.13	108.99	0.447	11	Vacuole
g3924.t1	*AtrKEA1*	1217	131.97	5.02	44.9	103.8	0.02	10	Nucleus
g7796.t1	*AtrKEA2*	795	86.19	5.28	37.74	114.92	0.331	0	Plasma Membrane
g11419.t1	*AtrKEA3*	1250	135.36	6.57	40.14	106.29	0.299	10	Chloroplast
g11543.t1	*AtrKEA4*	435	47.69	6.82	39.54	121.7	0.637	8	Plasma Membrane
g22883.t1	*AtrKEA5*	582	63.81	5.72	28.64	116.72	0.568	10	Plasma Membrane
g784.t1	*AtrCPA2-1*	826	90.05	6.08	39.08	114.25	0.346	12	Vacuole
g1479.t1	*AtrCPA2-2*	673	74.59	5.57	35.87	111.34	0.35	8	Vacuole
g3652.t1	*AtrCPA2-3*	808	89.60	8.71	40.42	114.13	0.287	12	Vacuole
g4917.t1	*AtrCPA2-4*	802	86.24	7.58	33.28	110.39	0.387	10	Plasma Membrane
g5517.t1	*AtrCPA2-5*	762	85.31	8.94	36.32	102.17	0.178	11	Plasma Membrane
g6141.t1	*AtrCPA2-6*	711	77.63	7.23	39.87	105.4	0.3	8	Plasma Membrane
g10210.t1	*AtrCPA2-7*	222	25.08	5.75	23.57	109.14	0.194	1	Nucleus
g10238.t1	*AtrCPA2-8*	770	85.94	9.21	38.62	102.48	0.226	10	Plasma Membrane
g10240.t1	*AtrCPA2-9*	806	89.34	8.97	44.39	115.09	0.34	9	Plasma Membrane
g11973.t1	*AtrCPA2-10*	774	85.26	7.84	31.56	109.56	0.359	10	Plasma Membrane
g13265.t1	*AtrCPA2-11*	279	30.96	7	36.97	132.08	0.907	6	Plasma Membrane
g13271.t1	*AtrCPA2-12*	811	90.62	6.42	30.28	111.37	0.406	10	Plasma Membrane
g15229.t1	*AtrCPA2-13*	787	88.43	5.79	36.95	107.85	0.311	9	Plasma Membrane
g15998.t1	*AtrCPA2-14*	842	92.27	7.6	38.75	108.97	0.263	9	Plasma Membrane
g17069.t1	*AtrCPA2-15*	827	90.98	5.9	45.8	115.94	0.355	12	Plasma Membrane
g18403.t1	*AtrCPA2-16*	800	86.72	8.57	37.7	109.31	0.388	10	Plasma Membrane
g19386.t1	*AtrCPA2-17*	422	47.49	4.58	37.88	108.29	0.22	5	Plasma Membrane
g22241.t1	*AtrCPA2-18*	779	85.98	8.33	27	114.39	0.371	10	Plasma Membrane
g23418.t1	*AtrCPA2-19*	789	89.82	9.02	39.21	111.72	0.285	11	Plasma Membrane
g25711.t1	*AtrCPA2-20*	280	31.26	9.47	27.61	114.82	0.474	6	Plasma Membrane
g26069.t1	*AtrCPA2-21*	791	87.75	8.11	29.87	104.84	0.277	11	Plasma Membrane

**Table 2:** Quantitative primer sequences for *AtrNHX* genes.

Gene Name	Forward Primer Sequences (5′-3′)	Reverse Primer Sequences (5′-3′)
*AtrNHX1*	TGTCATTGCCCAAGGTGT	TTCTCTCCAGTCCAAACCAT
*AtrNHX2*	CCAGGGCTGTGAATGTGTT	CCAGGGCTGTGAATGTGTT
*AtrNHX3*	CAGAAACAAGCATCAGGGA	AAAGTGAGGCTATGAAGGTCC
*AtrNHX4*	TCATTTACTTGCTGCCACC	GCTGCGTCAATCCAATCTT
*AtrNHX5*	GCTTTCGCAACACTGTCTTT	CAACCATCACTAAACCGAGC
*AtrNHX6*	ATGCTTGCCGAACTCTTC	TCTCAATGTCTAATGCGTCC
*AtrNHX7*	TGAGGAAGATTGGTTTGACG	AGGTCGCATCATTCACTACTC
*AtrNHX8*	GGGAAGGTGTTGTAAATGATGC	TCCTGTCAGAACTCCGAGAATG
*AtrNHX9*	CGTGTTTCCTTTATCCGC	CATCGTAGCATTGACAGTATCC
*AtrEF1a*	GGGATGCTGGTATGGTGAA	ACGGGTCATTTCTTCTTCTGAG

**Table 3:** PCR and qRT-PCR primer design for transgenic *Arabidopsis thaliana*.

Gene	Forward Primer Sequences (5′-3′)	Reverse Primer Sequences (5′-3′)	Usage
*AtrNHX8*	CGGGGTACCATGCGATGAAAGTGCCTTT	ACGCGTCGACCCTATTACTGAAGTAGTCTAC	Vector construction
*GUS*	ACGTCCTGAAGAAACCCCAACC	TCCCGGCAATAACATACGGCGT	PCR
*Hyg*	CTATTTCTTTGCCCTCGGACGAG	GAATCGGTCAATACACTACATGGC	PCR
*AtNHX4*	CTTGACTGTGTTCTTCTGCG	CAACGTCCCATTTCTCGAT	qRT-PCR

## Data Availability

All datasets generated for this study are included in the article.

## Supplementary Materials

The following supporting information can be downloaded at: https://www.mdpi.com/article/10.3390/plants13121701/s1, Figure S1: Analysis of transmembrane structure in *Amaranthus tricolor*.
