# Supplementary figures and images for: Genome-Wide Identification of the Cation/Proton Antiporter (CPA) Gene Family and Functional Analysis of AtrNHX8 under Salt Stress
Source: Plants (Basel). 2024 Jun 19;13(12):1701. doi: 10.3390/plants13121701 (PMC11207833; doi:10.3390/plants13121701)

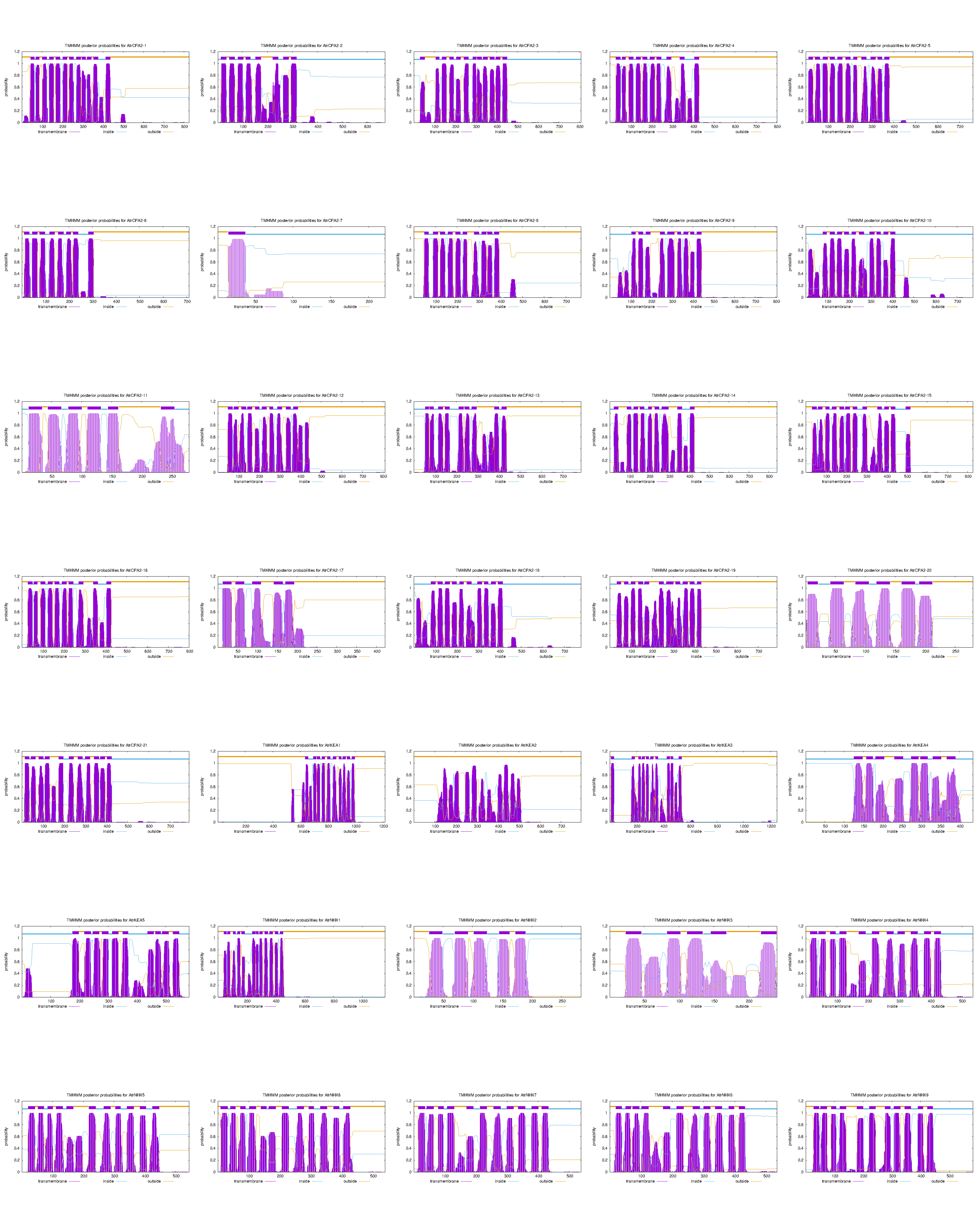

Supplement: Supplementary file 1 [file plants-13-01701-s001.zip › plants-3023850-supplementary.tif]
